# Effects of Digital Cognitive Rehabilitation on Functional Performance After Stroke: A Systematic Review and Meta-Analysis of ADL and IADL Outcomes

**DOI:** 10.3390/healthcare14152355

**Published:** 2026-08-02

**Authors:** Jae Hun Jung

**Affiliations:** Department of Occupational Therapy, Kaya University, Gimhae 50830, Republic of Korea; otjjh@kaya.ac.kr

**Keywords:** digital cognitive rehabilitation, functional performance, stroke, functional cognition, activities of daily living, instrumental activities of daily living, meta-analysis

## Abstract

**Background/Objectives:** Digital cognitive rehabilitation (DCR) is increasingly used for post-stroke cognitive rehabilitation, but its effects on functional performance remain insufficiently synthesized. This review examined the effects of DCR on activities of daily living (ADL) and instrumental activities of daily living (IADL) after stroke. **Methods:** This review followed the PRISMA 2020 statement and was registered in PROSPERO. Five bibliographic databases and Google Scholar were searched for randomized controlled trials published between 2010 and 2025. Methodological quality, risk of bias, and certainty of evidence were assessed using the PEDro scale, Cochrane RoB 2 tool, and GRADE framework. Effect sizes were calculated as Hedges’ g and pooled using random-effects models. **Results:** Eighteen RCTs were included in the systematic review. No included study directly assessed functional cognition. PEDro scores ranged from 4 to 8, and most studies were rated as having “some concerns” for overall risk of bias. DCR showed statistically significant moderate effects on ADL performance (k = 12, N = 503; Hedges’ g = 0.463, 95% CI = 0.241–0.684, *p* < 0.001; I^2^ = 36.73%) and IADL performance (k = 4, N = 186; Hedges’ g = 0.580, 95% CI = 0.248–0.912, *p* = 0.001; I^2^ = 23.49%). No statistically significant subgroup differences or meta-regression associations were detected for ADL outcomes, but these exploratory analyses were limited by the small number of studies. The certainty of evidence was moderate for ADL outcomes and low for IADL outcomes. **Conclusions:** DCR may improve ADL performance after stroke. IADL effects were also significant, but the evidence for IADL outcomes remains limited. Prediction intervals for both outcomes indicated uncertainty about the effects that may be observed in future trials. Because no included study directly assessed functional cognition, these findings should be interpreted as evidence on ADL and IADL functional performance outcomes, not as direct evidence of effects on functional cognition. Clinical application should consider methodological limitations, risk-of-bias concerns, and certainty of evidence. Future RCTs should include validated performance-based measures of functional cognition.

## 1. Introduction

Recovery of functional performance is considered a central goal of post-stroke rehabilitation [1]. Functional performance refers to the ability to independently perform meaningful activities in real-world contexts and reflects an individual’s capacity for independent living and social participation [2]. It is commonly represented by activities of daily living (ADL) and instrumental activities of daily living (IADL) [3]. ADL includes basic self-care activities such as eating, dressing, and personal hygiene, whereas IADL involves more complex activities that require cognitive processing and executive abilities, such as meal preparation and cleanup, financial management, and shopping [4]. Therefore, functional performance is an important indicator of independent daily living and community reintegration after stroke and is closely associated with various cognitive functions [5].

Cognitive impairment has been reported as a major factor limiting functional performance after stroke [6]. In particular, executive function is a core cognitive domain that supports goal-directed behavior, planning, and problem solving and is closely related to the performance daily activities [7]. Other cognitive functions, such as attention and memory, also provide the foundation for activity performance. Impairments in these functions may affect not only complex everyday tasks but also basic self-care activities, including eating, personal hygiene, and dressing [8]. Consequently, cognitive impairment can reduce independence across ADL and IADL, and cognitive rehabilitation has been used as an important intervention to support functional recovery after stroke [9].

Previous cognitive rehabilitation studies have primarily relied on neuropsychological test-based outcomes, and have therefore tended to emphasize changes in cognitive function [10,11]. In contrast, assessments that reflect cognitive function directly related to real-life performance have been limited. A previous review reported that functional performance outcomes were included in some cognitive rehabilitation studies, but they were often treated as secondary outcomes, with the primary focus remaining on cognitive improvement [12]. This suggests that cognitive rehabilitation research has not sufficiently incorporated assessments that reflect real-life performance contexts.

In response to this limitation, functional cognition has become increasingly emphasized in cognitive rehabilitation [13]. Functional cognition refers to the use of cognitive abilities during everyday activity performance, as shaped by the interaction among task demands, environmental contexts, and personal factors [14]. These cognitive processes are reflected in observable behaviors during everyday task performance [15]. Thus, functional cognition provides important information for understanding the relationship between cognitive function and real-world performance [16].

At the same time, advances in digital technology have expanded the ways in which cognitive rehabilitation can be delivered after stroke. DCR has been applied in various forms, and an increasing number of studies have examined its effects [17,18,19]. DCR has gained attention because it can provide repetitive and structured cognitive training through various technologies, including virtual reality (VR), computer-based cognitive training, mobile applications, and telerehabilitation programs [20,21]. In particular, these approaches may enable task-oriented training by simulating environments similar to real-life situations or by incorporating functional components into the intervention [22]. However, despite these technological advances, the diversity of intervention types, intervention components, and intervention dosage continues to make it difficult to interpret findings consistently.

Several systematic reviews and meta-analyses have attempted to synthesize the effects of DCR. However, several issues remain to be addressed. First, many previous reviews have focused on specific technology types, such as VR or computer-based cognitive training [23,24,25,26,27]. This technology-specific focus has limited the extent to which DCR has been understood as an integrated intervention concept [28,29]. Although DCR interventions may differ in technological platforms, they share the core feature of delivering cognitive rehabilitation or cognitive training through digital technologies. Second, previous reviews have primarily emphasized neuropsychological test-based outcomes, while the functional cognition perspective, which reflects the use of cognition in real-life performance contexts, has not been sufficiently considered [29,30]. Third, although some reviews have included functional performance outcomes such as ADL and IADL, these outcomes have often been treated as secondary outcomes [23,27,31,32]. In addition, heterogeneity in outcome measures and intervention characteristics remains an important issue when synthesizing functional performance outcomes across studies [28,29,30]. These issues limit a comprehensive understanding of the effects of DCR on functional performance in real-life contexts.

Considering these issues, there is a need to examine DCR as an integrated intervention concept rather than as a set of individual technology-specific approaches. It is also necessary to evaluate its effects from a functional performance perspective, with attention to performance-based outcomes that reflect the use of cognition in real-life contexts. Therefore, this systematic review and meta-analysis aimed to examine the effects of DCR on functional performance, particularly ADL and IADL, in individuals after stroke. By synthesizing the available evidence, this review sought to provide a basis for evidence-based practice aimed at improving functional performance in post-stroke cognitive rehabilitation.

## 2. Materials and Methods

### 2.1. Study Design and Registration

This study synthesized evidence on the effects of DCR on functional performance among adults with post-stroke cognitive impairment. The review process and reporting followed the Preferred Reporting Items for Systematic Reviews and Meta-Analyses (PRISMA) 2020 statement [33]. The protocol was registered prospectively in PROSPERO before conducting the review (CRD420251182488; registered on 3 November 2025). The review question and planned analyses were derived from the prespecified protocol.

### 2.2. Eligibility Criteria

Eligible studies were identified according to the Population, Intervention, Comparison, Outcomes, and Study design (PICOS) framework. Studies were included if they investigated DCR in relation to functional performance outcomes among adults with post-stroke cognitive impairment.

#### 2.2.1. Population

Eligible participants were adults aged 18 years or older with cognitive impairment following ischemic or hemorrhagic stroke. Studies were excluded if they focused on neurological conditions other than stroke, included participants younger than 18 years, or used animal models.

#### 2.2.2. Intervention

DCR was defined as a digital or technology-assisted cognitive rehabilitation intervention. Eligible interventions included cognitive training programs delivered through technology-mediated approaches, such as VR-based training, computer-based cognitive training, mobile applications, serious games, web-based programs, or telerehabilitation. Although these interventions differed in technological platform, eligibility was based on the presence of a digital cognitive training component rather than on a specific technology type. To be included, interventions had to contain structured, repetitive, and goal-oriented cognitive training components aimed at improving cognitive functions such as attention, memory, or executive function. Studies in which DCR was combined with conventional rehabilitation, such as occupational therapy or physical therapy, were also included if the digital cognitive training component was clearly identifiable. Studies were excluded if the intervention consisted solely of paper-and-pencil cognitive training without a technological interface or focused on physical function, balance, or motor rehabilitation without a cognitive training component.

#### 2.2.3. Comparison

Eligible comparators included usual care, conventional rehabilitation, or cognitive interventions that did not involve digital technology. Waitlist controls and passive control groups that received no additional intervention were also included.

#### 2.2.4. Outcomes

In accordance with the protocol registered in PROSPERO, functional cognition was prespecified as the primary outcome. The protocol also included a prespecified fallback rule stating that, if fewer than three eligible RCTs reported functional cognition outcomes, ADL and IADL outcomes would be analyzed as primary outcomes, and functional cognition outcomes, if any, would be narratively synthesized.

#### 2.2.5. Study Design

The review was limited to randomized controlled trials (RCTs). Non-randomized studies, single-group pre–post studies, case studies, qualitative studies, conference abstracts, and review articles were excluded. Studies that did not provide sufficient statistical data for meta-analysis were included in the systematic review but excluded from the quantitative synthesis.

### 2.3. Information Sources and Search Strategy

A systematic literature search was conducted using CINAHL, Cochrane Library, Embase, PsycARTICLES, PubMed, and Google Scholar. The search was limited to studies published in English between 1 January 2010 and 30 December 2025. The final search was conducted on 16 January 2026. For Google Scholar, the first 200 records for each search strategy were screened, consistent with previous recommendations for managing Google Scholar searches in systematic reviews [34].

The search strategy was developed around the main concepts relevant to post-stroke cognitive rehabilitation. These concepts included stroke, cognitive rehabilitation, digital or technology-based interventions, and functional performance outcomes. Terms related to performance-based assessments of functional cognition were also incorporated into the search strategy. These terms included instruments such as the Executive Function Performance Test (EFPT) and the Weekly Calendar Planning Activity (WCPA), which were classified as functional cognition assessments in a previous systematic review [16].

Search terms were combined using Boolean operators (AND and OR) and adapted to the syntax and indexing structure of each database. The full search strategies for each database are provided in Supplementary Table S1.

### 2.4. Study Selection

Study selection was conducted independently by two reviewers (S.R. and J.Y.) according to the predefined eligibility criteria. Records retrieved from the database searches were managed in EndNote version 21 (Clarivate, Philadelphia, PA, USA). Duplicate records were first removed using the automatic duplicate detection function in EndNote and were then additionally checked manually. The two reviewers then screened the titles and abstracts of the remaining records to identify potentially eligible studies. The full-text articles of potentially eligible studies were subsequently retrieved and assessed by the two reviewers to determine final inclusion. Any disagreements during study selection were discussed with the author (J.H.) until consensus was reached.

### 2.5. Data Extraction

Data extraction was performed independently by two reviewers (S.R. and J.Y.) using a predefined data extraction form. Extracted data were organized in Microsoft Excel for Microsoft 365 (Microsoft Corporation, Redmond, WA, USA). The extracted information included general study characteristics, participant characteristics, intervention characteristics, comparison condition, and outcome measures. Specifically, the following data were extracted: author, year of publication, country, study design, study setting, sample size for each group, participant age, intervention, intervention dosage, comparison condition, and outcome measures.

To systematically describe the characteristics of DCR interventions, additional information related to intervention structure and dosage was extracted, including intervention, technology approach, device, combined intervention, functional component, delivery mode, session length, weekly frequency, and intervention duration. The total number of sessions and total training hours were calculated from the extracted intervention dosage information when sufficient information was available. The intervention category was classified according to the core DCR component described in each study. The technology approach was classified according to the technological format through which the intervention was implemented. Devices were classified according to the physical equipment used to deliver the intervention, allowing interventions with the same technology approach to be distinguished by the device used. A combined intervention was defined as a DCR intervention delivered together with another therapeutic component. A functional component was defined as the inclusion of activities involving ADL tasks or activities resembling real-life contexts. Any discrepancies between the two reviewers during data extraction were resolved through discussion with the author (J.H.).

### 2.6. Methodological Quality, Risk of Bias, and Certainty of Evidence

Methodological quality, risk of bias, and certainty of evidence were assessed independently by two reviewers (S.R. and J.Y.). Any discrepancies between the two reviewers during these assessments were resolved through discussion with the author (J.H.).

Methodological quality was assessed using the Physiotherapy Evidence Database (PEDro) scale, which is widely used in physical therapy and rehabilitation research. The PEDro scale consists of 11 items: eligibility criteria, random allocation, allocation concealment, baseline comparability, blinding of subjects, blinding of therapists, blinding of assessors, adequate follow-up, intention-to-treat analysis, between-group comparisons, and point estimates and variability [35]. The eligibility criterion item is not included in the total score. Therefore, the total PEDro score ranges from 0 to 10. Each item was scored as 0 or 1 according to whether the criterion was satisfied [36].

Risk of bias was assessed using the Cochrane Risk of Bias 2 (RoB 2) tool. This tool evaluates five domains: bias arising from the randomization process, bias due to deviations from the intended interventions, bias due to missing outcome data, bias in measurement of the outcome, and bias in selection of the reported result. Each domain was rated as “low risk”, “some concerns”, or “high risk” based on the signaling questions, and an overall risk-of-bias judgment was then determined [37].

The certainty of evidence was assessed using the Grading of Recommendations Assessment, Development and Evaluation (GRADE) approach. GRADE evaluates the certainty of evidence for each outcome across five domains: risk of bias, inconsistency, indirectness, imprecision, and publication bias. The certainty of evidence was rated as “high”, “moderate”, “low”, or “very low” [38]. In this review, GRADE was applied only to outcomes included in the meta-analysis, using the web-based GRADEpro GDT software [39].

### 2.7. Data Synthesis and Statistical Analysis

Meta-analysis was performed to quantitatively synthesize the findings of the included studies. Statistical analyses were conducted using Comprehensive Meta-Analysis (CMA), version 4.0 (Biostat, Englewood, NJ, USA). Outcomes that were not eligible for meta-analysis were summarized using narrative synthesis. Outcome synthesis was conducted in accordance with the prespecified fallback rule in the prospectively registered PROSPERO protocol, as described in Section 2.2.4. Under this framework, ADL and IADL outcomes were analyzed as distinct outcome domains of functional performance. When a study reported both ADL and IADL outcomes, each outcome was extracted as a separate effect size and included in the corresponding analysis. Because the included studies used different outcome measures, effect sizes were calculated as standardized mean differences (SMDs) using Hedges’ g.

Effect sizes were calculated using post-treatment outcome data. When multiple post-baseline time points were reported, the assessment conducted immediately after the intervention period was selected for the primary meta-analysis. For most studies, Hedges’ g was calculated from post-treatment means, standard deviations, and sample sizes for the intervention and control groups. When post-treatment means and standard deviations were not available, effect sizes were calculated from other post-treatment statistics, such as reported standardized differences in means or t values, when sufficient information was available. Change scores were not used because they were not consistently available across studies. All effect sizes for the meta-analyzed ADL and IADL outcomes were coded in the same direction, with positive values indicating more favorable performance in the DCR group than in the control group. Effect sizes were reported with 95% confidence intervals (CIs). For multi-arm studies included in the meta-analysis, one intervention arm and one comparator arm were selected from each study to avoid double-counting participants. When multiple intervention arms were available, the intervention arm most closely aligned with the review question was selected. When multiple comparator arms were available, the conventional or non-DCR comparator arm most appropriate for the selected intervention contrast and review question was selected, where applicable. Therefore, each multi-arm study contributed only one pairwise comparison to the meta-analysis.

Studies were excluded from the meta-analysis if they did not provide sufficient statistical data for effect size calculation. In particular, studies reporting only medians with interquartile ranges or minimum–maximum ranges were not converted into means and standard deviations because such conversions require additional distributional assumptions and may introduce uncertainty into standardized effect size estimates [40,41]. Although excluded from the quantitative synthesis, these studies were retained in the systematic review and summarized narratively. Effect sizes were interpreted according to Cohen’s criteria, with values of 0.2, 0.5, and 0.8 indicating small, moderate, and large effects, respectively [42].

A random-effects model was prespecified to account for expected clinical and methodological heterogeneity across studies. This decision reflected the possibility that the true intervention effects could differ across studies because of variation in DCR type, intervention setting, and outcome measures [43]. Within this framework, the pooled effect estimates were interpreted as overall estimates across eligible DCR interventions on ADL and IADL outcomes. Between-study heterogeneity was examined with Cochran’s Q and quantified using I^2^. A threshold of *p* < 0.10 was used for Cochran’s Q because the test has limited power when few studies are included [44]. I^2^ values were interpreted as low at approximately 25%, moderate at 50%, and high at 75% [45].

Subgroup analyses and meta-regression analyses were conducted to explore potential moderators of the intervention effect, considering both statistical heterogeneity and clinical or methodological differences across studies. Subgroup analyses were based on prespecified categories and were finalized according to the characteristics of the included studies and data availability. The subgroup analyses examined functional component inclusion, combined intervention, and comparison type. Functional component was categorized as included versus not included. Combined intervention status was categorized as DCR alone, also referred to as stand-alone DCR, versus DCR combined with another intervention. DCR alone was defined as an intervention in which DCR was delivered without another intervention as part of the intervention package. DCR combined with another intervention was defined as an intervention in which DCR was delivered together with another intervention as part of the intervention package. Comparison type as active versus passive. Meta-regression analyses were conducted to examine the associations between intervention characteristics and effect sizes. The following continuous variables were included: session length, weekly frequency, intervention duration, total number of sessions, and total training hours.

The stability of the pooled estimates was examined through sensitivity analyses. Leave-one-out analysis was conducted to determine whether the overall effect size was influenced by any single study [46]. To investigate potential publication bias, a visual assessment was conducted using funnel plot asymmetry, complemented by a statistical evaluation through Egger’s regression test [47].

## 3. Results

### 3.1. Study Selection

**Figure 1:**
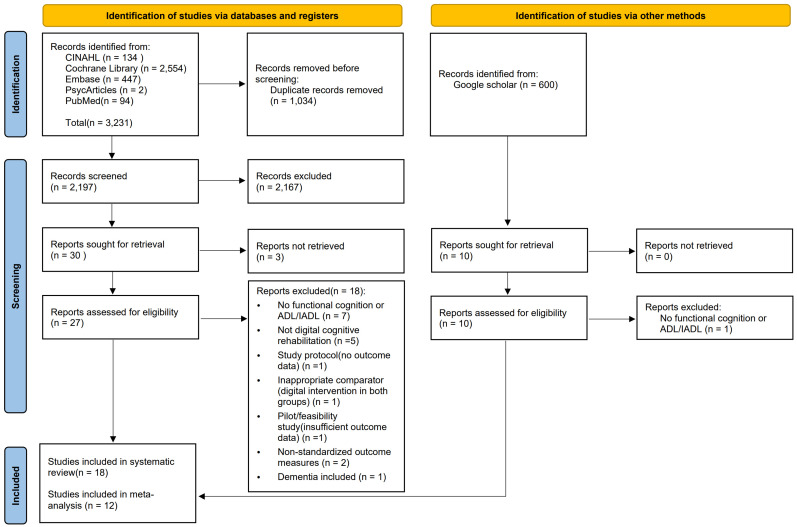
PRISMA 2020 flow diagram of study selection.

Database searches retrieved 3231 records in total (CINAHL = 134, Cochrane Library = 2554, Embase = 447, PsycARTICLES = 2, and PubMed = 94). An additional 600 records were identified through Google Scholar. After removing 1034 duplicate records from the database search results, 2197 records underwent title and abstract screening. At this stage, 2167 records were excluded, and 30 reports were selected for retrieval. Three reports were unavailable, leaving 27 reports for eligibility assessment. Among the reports identified through database searches, 18 were excluded after full-text assessment. The reasons for exclusion were as follows: no functional cognition or ADL/IADL outcomes reported (*n* = 7), not a DCR intervention (*n* = 5), study protocol with no outcome data (*n* = 1), inappropriate comparison condition because digital interventions were provided in both groups (*n* = 1), pilot or feasibility study with insufficient outcome data (*n* = 1), use of non-standardized outcome measures (*n* = 2), and inclusion of participants with dementia (*n* = 1). Among the records identified through Google Scholar, 10 reports were sought for retrieval and assessed for eligibility. Of these, one report was excluded because it did not report functional cognition or ADL/IADL outcomes. In total, 18 studies were included in the systematic review, and 12 of these were selected for the meta-analysis. None of the included studies directly assessed functional cognition. Therefore, in accordance with the prespecified fallback rule in the prospectively registered PROSPERO protocol, ADL and IADL outcomes were analyzed as the main functional performance outcomes in this review. The PRISMA 2020 flow diagram summarizes the study selection process (Figure 1).

### 3.2. Study Characteristics

A total of 18 RCTs were included in the systematic review [48,49,50,51,52,53,54,55,56,57,58,59,60,61,62,63,64,65]. A summary of the characteristics of the studies included can be found in Table 1. Of these, 15 studies were published after 2020. Most studies were conducted in hospital-based settings, including inpatient or outpatient rehabilitation settings. Only a few studies were carried out in settings such as homes or rehabilitation clinics [60,61]. The included RCTs were distributed across several countries. Many of the studies were predominantly conducted in East Asian regions, including countries such as China, Korea, and Taiwan. Other studies were conducted in Portugal, Italy, India, and Iran. The total number of participants across the included studies was 838, and sample sizes ranged from 18 to 72 participants. Mean ages ranged from approximately 49 to 76 years. Most studies reported age as mean and standard deviation, whereas some studies [51,52,55,64] reported median and interquartile range. Detailed information on cognitive impairment criteria or diagnoses, stroke type, time since stroke, stroke severity, and baseline cognitive and functional status is provided in Supplementary Table S2. When reported, cognitive impairment was identified using screening criteria based on the Mini-Mental State Examination (MMSE), Montreal Cognitive Assessment (MoCA), or Korean Mini-Mental State Examination, second edition (K-MMSE-2), or using study-specific diagnostic categories such as post-stroke cognitive impairment (PSCI) or vascular cognitive impairment (VCI). Participant characteristics that were not reported in the original studies were recorded as not reported.

The included studies applied various types of DCR interventions. The characteristics of the interventions are detailed in Table 2 and are further explained in the subsequent section. Multi-arm designs were used in seven studies [48,49,50,56,59,62,64]. Intervention duration ranged from 2 to 12 weeks. Most studies assessed outcomes immediately after the intervention, whereas some studies [48,53,54] additionally conducted follow-up assessments to examine the maintenance of intervention effects. Comparison conditions included active controls, such as conventional cognitive training or conventional occupational therapy, and passive controls, such as usual care, waitlist, or no intervention.

Various standardized outcome measures were used to assess functional performance. ADL was assessed using measures such as the Functional Independence Measure (FIM), Barthel Index (BI or MBI), Korean Modified Barthel Index (K-MBI), and Stroke Impact Scale–ADL (SIS-ADL). IADL was assessed using the Lawton IADL scale in three studies [60,62,64] and the Luton Index in one study [61]. Veisi-Pirkoohi et al. [61] described the Luton Index as an interview-based IADL measure assessing eight daily activities: telephone use, shopping, food preparation, housekeeping, laundry, transportation, responsibility for medication use, and financial activities. Each item was scored 0 or 1, with total scores ranging from 0 to 8. One study [48] assessed both ADL and IADL using the Adults and Older Adults Functional Assessment Inventory (IAFAI). ADL and IADL were assessed separately in four studies [60,61,62,64].

### 3.3. Intervention Characteristics

The characteristics of the DCR interventions are presented in Table 2. For studies with a multi-arm design, all intervention arms that included a DCR component were presented separately.

The included interventions varied in program type and intervention structure. Most interventions were based on computer-based cognitive training, including both commercially available programs and researcher-developed programs. In terms of technology approach, computer-based interventions were the most common, followed by VR, AR, mobile application-based, and other digital approaches. Regarding device type, PCs and tablets were frequently used, whereas VR-based interventions used equipment such as head-mounted displays or goggles. Of the 23 intervention arms, 10 delivered DCR in combination with another intervention, such as transcranial direct current stimulation (tDCS), conventional occupational therapy, aerobic exercise, prism adaptation, workbooks, BADL training, or conventional USN training. Functional components were identified in seven intervention arms. In some studies, the intervention was primarily composed of functional tasks [48,51], whereas in others, functional content was partially embedded within a cognitive training structure [52,57,58]. In one study, the functional component was included as part of a combined intervention [64].

Most interventions were delivered under therapist supervision. One study used a remotely delivered format with periodic therapist monitoring [60]. Intervention dosage varied across studies. Session length ranged from approximately 15 to 90 min, weekly frequency ranged from 2 to 6 sessions per week, and intervention duration ranged from 2 to 12 week. 

### 3.4. Methodological Quality and Risk of Bias

#### 3.4.1. Methodological Quality

The methodological quality evaluation of the 18 studies, conducted using the PEDro scale, is summarized in Table 3. Total PEDro scores ranged from 4 to 8. Random allocation was performed in all studies. Allocation concealment was confirmed in five studies, and baseline comparability was achieved in 16 studies. Blinding of subjects and blinding of therapists was not implemented in any study, whereas blinding of assessors was confirmed in 11 studies. Adequate follow-up criteria were met in 14 studies, and intention-to-treat analysis was conducted in 12 studies. Between-group comparisons were presented in 17 studies, and point estimates and measures of variability were provided in 16 studies.

#### 3.4.2. Risk of Bias

**Figure 2:**
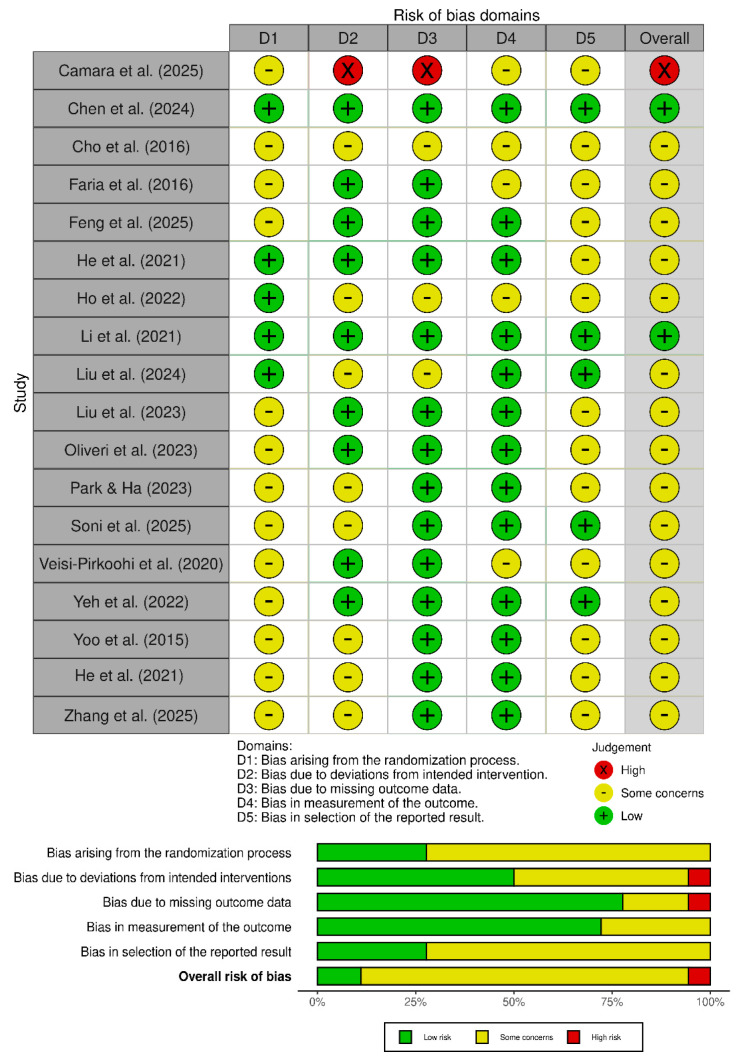
Risk of bias assessment of the included studies [48,49,50,51,52,53,54,55,56,57,58,59,60,61,62,63,64,65].

Risk of bias in the included studies was assessed using the RoB 2 tool, and the results are presented in Figure 2. In the overall judgment, most studies were rated as having “some concerns.” Two studies were rated as “low risk”, and one study was rated as “high risk.”

For individual RoB 2 domains, approximately 72% of studies were rated as having “some concerns” for bias arising from the randomization process (D1) and bias in selection of the reported result (D5). For bias due to deviations from the intended interventions (D2), 50% of studies were rated as having “some concerns”. In contrast, approximately 78% of studies were rated as “low risk” for bias due to missing outcome data (D3), and approximately 72% were rated as “low risk” for bias in measurement of the outcome (D4).

### 3.5. Synthesis of Functional Performance Outcomes

Meta-analysis was conducted for studies that met the prespecified criteria for effect size calculation. Functional performance outcomes were synthesized separately for ADL and IADL. The ADL meta-analysis included 12 studies, whereas four studies contributed data to the IADL meta-analysis. The quantitative findings for ADL and IADL are presented separately in the following sections.

Six studies were excluded from the meta-analysis because they did not provide sufficient statistical information for effect size calculation [48,49,51,53,55,56]. These studies lacked sufficient statistical information because functional performance outcomes were reported as medians with interquartile ranges or minimum–maximum ranges, or were presented graphically without exact numerical values. Although excluded from the meta-analysis, these studies remained part of the systematic review, and their functional performance outcomes were summarized narratively.

Among the narratively summarized studies, three reported ADL outcomes [51,53,55], two reported IADL outcomes [49,56], and one reported a functional performance outcome covering both ADL and IADL components [48]. For ADL outcomes, one study reported statistically significant findings favoring the intervention condition [53], one study found no significant between-group difference [51], and one study reported significant within-group improvements in both groups without a clear between-group advantage for ADL performance [55]. For IADL outcomes, one study reported statistically significant findings favoring the combined intervention condition [49], whereas one study found no significant between-group difference [56]. The study using a functional performance measure covering both ADL and IADL components reported statistically significant improvement favoring the intervention compared with the control group [48]. These six studies are summarized in Supplementary Table S3.

### 3.6. Meta-Analysis of ADL Outcomes

#### 3.6.1. Overall Effect

**Figure 3:**
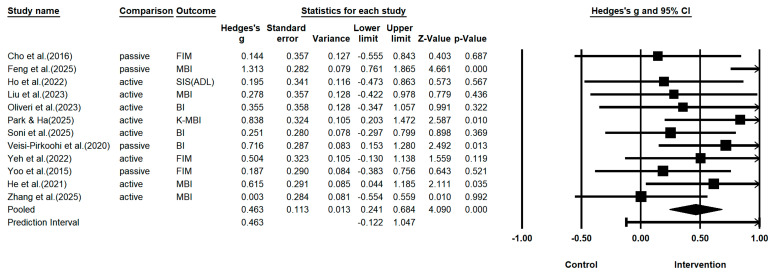
Forest plot of the effects of digital cognitive rehabilitation on ADL outcomes [50,52,54,57,58,59,60,61,62,63,64,65].

The meta-analysis of ADL outcomes included 503 participants from 12 studies [50,52,54,57,58,59,60,61,62,63,64,65]. In the random-effects model, DCR was associated with a significant improvement in ADL performance, with a moderate effect size (Hedges’ g = 0.463, 95% CI = 0.241–0.684, *p* < 0.001). Low-to-moderate heterogeneity was observed between studies (Q = 17.387, df = 11, *p* = 0.097, I^2^ = 36.73%). However, the 95% prediction interval ranged from −0.122 to 1.047 and included no effect (Figure 3).

#### 3.6.2. Subgroup Analysis

Due to the observed low-to-moderate heterogeneity in ADL outcomes, subgroup analyses were performed to explore potential sources of heterogeneity and variations in effect sizes based on clinical and methodological characteristics (Table 4).

In the analysis according to functional component inclusion, studies with a functional component showed a larger effect size (Hedges’ g = 0.697, 95% CI = 0.334–1.060, *p* < 0.001) than studies without a functional component (Hedges’ g = 0.353, 95% CI = 0.102–0.604, *p* = 0.006). However, the difference between the subgroups did not reach statistical significance (Q = 2.344, *p* = 0.126).

In the analysis according to combined intervention status, comparisons classified as DCR combined with another intervention (Hedges’ g = 0.455, 95% CI = 0.093–0.816, *p* = 0.014) and those classified as DCR alone (Hedges’ g = 0.466, 95% CI = 0.163–0.770, *p* = 0.003) showed similar effect sizes. The between-subgroup difference was not statistically significant (Q = 0.002, *p* = 0.963).

In the analysis according to comparison type, studies using passive controls showed a larger effect size (Hedges’ g = 0.626, 95% CI = 0.256–0.996, *p* = 0.001) than studies using active controls (Hedges’ g = 0.376, 95% CI = 0.106–0.646, *p* = 0.006). However, the between-subgroup difference was not statistically significant (Q = 1.142, *p* = 0.285). Overall, no statistically significant between-subgroup differences were detected for any subgroup variable. Given the small number of studies within each subgroup, these subgroup analyses should be regarded as exploratory.

#### 3.6.3. Meta-Regression Analysis

Meta-regression analyses were performed to investigate the relationships between the dose-related features of DCR interventions and their effect sizes. The results are presented in Table 5. None of the examined variables was significantly associated with the effect size (*p* > 0.05). These variables included session length, weekly frequency, intervention duration, total number of sessions, and total training hours. Because the meta-regression analyses were based on only 12 studies and five continuous predictors were tested independently, statistical power was limited and false-negative findings cannot be ruled out. Therefore, these analyses should be regarded as exploratory.

#### 3.6.4. Sensitivity Analysis

**Figure 4:**
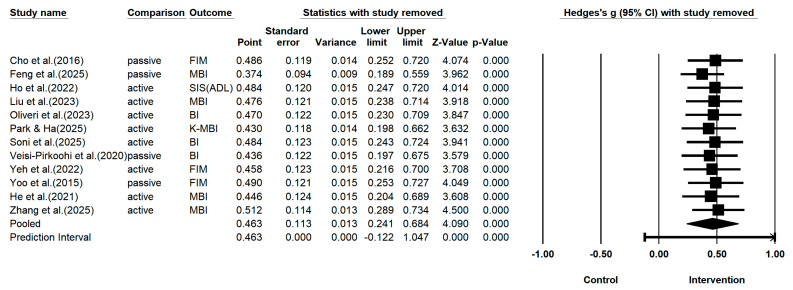
Leave-one-out sensitivity analysis for ADL outcomes [50,52,54,57,58,59,60,61,62,63,64,65].

The leave-one-out method was utilized to conduct the sensitivity analysis. By systematically excluding each study and conducting the analysis again, the pooled effect size ranged from Hedges’ g = 0.374 to 0.512. Statistical significance was maintained in all analyses. As part of this leave-one-out analysis, exclusion of Ho et al. [54], which used the SIS-ADL, yielded a pooled effect size similar to the overall ADL estimate (Hedges’ g = 0.484, 95% CI = 0.247–0.720, *p* < 0.001). The forest plot for the sensitivity analysis is presented in Figure 4.

#### 3.6.5. Publication Bias

**Figure 5:**
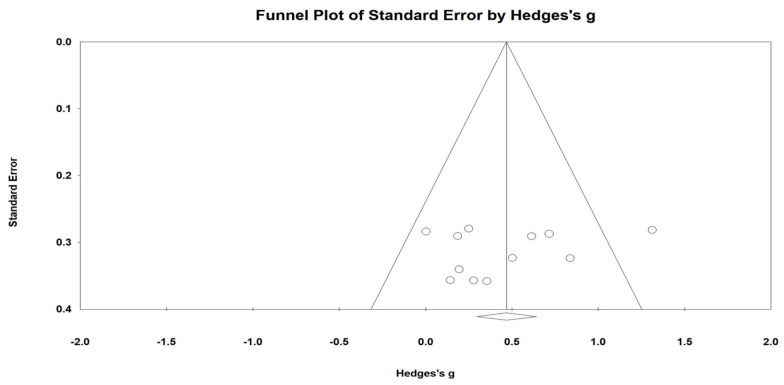
Funnel plot for publication bias of ADL outcomes.

Publication bias was evaluated using both a visual review of the funnel plot and Egger’s regression test. The visual analysis of the funnel plot pointed to a slight asymmetry (Figure 5). Egger’s regression test did not detect statistically significant asymmetry (*p* = 0.422). However, given the limited number of studies included in the ADL meta-analysis, the test may have had limited statistical power, and the possibility of publication bias cannot be completely excluded.

### 3.7. Meta-Analysis of IADL Outcomes

**Figure 6:**
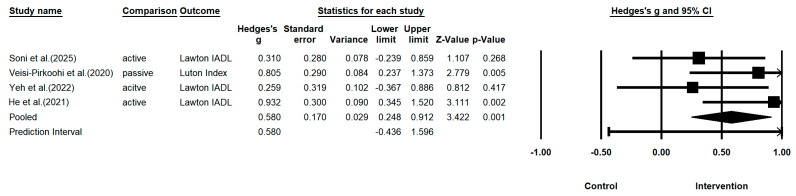
Forest plot of the effects of digital cognitive rehabilitation on IADL outcomes [60,61,62,64].

The meta-analysis of IADL outcomes included 186 participants from four studies [60,61,62,64]. DCR had a significant positive effect on IADL performance, with a moderate effect size (Hedges’ g = 0.580, 95% CI = 0.248–0.912, *p* = 0.001). Heterogeneity was low (Q = 3.912, df = 3, *p* = 0.270, I^2^ = 23.49%). However, the 95% prediction interval ranged from −0.436 to 1.596 and included no effect (Figure 6). A sensitivity analysis was conducted by excluding Veisi-Pirkoohi et al. [61], which reported the IADL outcome as the Luton Index rather than the Lawton IADL scale. The pooled IADL effect remained statistically significant, although the effect size was slightly reduced (Hedges’ g = 0.502, 95% CI = 0.080–0.924, *p* = 0.020; Supplementary Figure S1). Because only four studies reported IADL outcomes with sufficient data for quantitative synthesis, subgroup analysis, meta-regression, and publication bias assessment were not conducted for this outcome domain.

### 3.8. Certainty of Evidence for ADL and IADL Outcomes

The certainty of evidence was determined using the GRADE methodology, with the scope restricted to the meta-analyzed ADL and IADL outcomes. Because none of the included studies directly assessed functional cognition, no GRADE certainty rating was assigned for this outcome. Indirectness was not downgraded because the certainty assessment was based on the directly measured ADL and IADL outcomes included in the meta-analysis. The results are presented in Table 6.

The certainty of the evidence concerning ADL outcomes was evaluated as moderate. For the ADL outcome, all 12 studies included in the meta-analysis received an overall RoB 2 judgment of “some concerns”. This led to a one-level downgrade for risk of bias in the GRADE assessment. No additional downgrading was made for inconsistency, indirectness, imprecision, or publication bias. Imprecision was not downgraded because the 95% confidence interval did not include the null effect.

The level of certainty regarding the evidence for IADL outcomes was determined to be low. The four studies included in the IADL meta-analysis were judged as having “some concerns” for risk of bias, resulting in downgrading by one level. An additional one-level downgrade was applied for imprecision because of the limited number of studies and participants and the relatively wide confidence interval. No downgrading was applied for inconsistency, indirectness, or publication bias.

## 4. Discussion

This systematic review and meta-analysis investigated the effects of DCR on functional performance in individuals after stroke. Among the 18 RCTs included in the systematic review, DCR interventions varied in technology approach, device, intervention components, delivery mode, and intervention dosage. The meta-analysis indicated significant favorable effects of DCR on both ADL and IADL performance, with moderate effect sizes. Heterogeneity was low-to-moderate for ADL and low for IADL. However, the prediction intervals for both outcomes included no effect, indicating that future trials may show smaller, limited, or no benefits. Therefore, these findings should be interpreted cautiously and should not be taken to indicate that DCR will consistently improve ADL or IADL outcomes across all post-stroke populations or intervention contexts. Because not all included studies provided sufficient data for meta-analysis, the pooled estimates should be interpreted alongside the broader systematic review findings, including studies summarized narratively.

Another important consideration is that the pooled effects mainly reflect immediate post-treatment outcomes. Most included studies assessed ADL and IADL outcomes at the end of the intervention period. Therefore, the clinical importance and long-term durability of the observed effects remain uncertain. The present findings should not be interpreted as evidence that the benefits of DCR are sustained over time. Future RCTs should include follow-up assessments to determine whether improvements in ADL and IADL performance are maintained after the intervention period.

In this review, functional cognition was prespecified as the primary outcome. However, none of the included studies directly assessed functional cognition. Therefore, the effect of DCR on functional cognition itself could not be synthesized. Importantly, the prospectively registered PROSPERO protocol included a prespecified fallback rule for outcome synthesis. In accordance with this prespecified fallback rule, ADL and IADL outcomes were analyzed as functional performance outcomes. Thus, the ADL/IADL synthesis should be interpreted as a prespecified analysis of functional performance rather than as evidence on functional cognition itself. Moreover, ADL and IADL outcomes are multidetermined and may be influenced not only by cognitive function but also by motor recovery, balance, physical assistance, environmental support, and overall stroke severity. Therefore, the present ADL/IADL findings should be interpreted as effects on functional performance outcomes, not as direct evidence of effects on functional cognition.

Functional cognition is closely related to the performance of everyday tasks and has been emphasized as an important construct in the occupational therapy literature [13,14,15,16]. However, previous DCR studies [29,30], as well as the RCTs included in this review, have continued to focus primarily on neuropsychological outcomes, such as attention, memory, executive function, and global cognition. The reasons for the absence of direct functional cognition outcomes cannot be determined from this review alone. However, this gap may partly reflect differences in outcome priorities across research fields and the fact that functional cognition has not yet been sufficiently operationalized as an outcome in DCR trials. In addition, performance-based functional cognition assessments, such as the EFPT and WCPA, require direct observation of complex everyday task performance and may involve greater administration, scoring, and training demands than commonly used ADL measures such as the FIM or MBI. These practical demands may have limited the incorporation of functional cognition measures into technology-based cognitive rehabilitation trials. Therefore, future DCR trials should incorporate validated performance-based measures of functional cognition, alongside ADL and IADL outcomes, to more directly evaluate cognitively demanding everyday task performance.

The 18 RCTs included in this review showed that DCR was delivered using various technological approaches and devices, including computer-based cognitive training, VR, augmented reality (AR), and mobile applications. This suggests that DCR is not a single intervention defined by a specific technology, but rather an expanded rehabilitation approach that can be flexibly implemented through different technology approaches to achieve similar therapeutic goals [66,67]. DCR was also delivered together with conventional occupational therapy, aerobic exercise, tDCS, and other interventions in some studies. Separately, some interventions incorporated functional components. These characteristics suggest that DCR is more commonly used as a complementary strategy that enhances the structure and repetition of rehabilitation and extends its connection to real-life contexts, rather than as a replacement for conventional rehabilitation [24,67,68]. However, most interventions were delivered under therapist supervision in hospital-based settings, whereas home-based applications were limited. This indicates that most current DCR studies have been conducted in structured clinical settings.

In this review, DCR showed a significant effect on functional performance, particularly ADL performance. However, previous meta-analyses have reported inconsistent findings regarding the effects of digital rehabilitation approaches on ADL outcomes after stroke. In the present review, subgroup analysis by technological approach was not performed because only two studies in the ADL meta-analysis used VR-based interventions. Nevertheless, previous evidence suggests that ADL outcomes may vary even within the same technology category. For example, previous meta-analyses of VR-based interventions have reported divergent findings for ADL outcomes, including significant improvements in one review [23] and no significant effect in another [26]. A similar pattern has been observed in meta-analyses of computer-based cognitive training [25,27]. These findings suggest that technology approach alone may not sufficiently explain improvements in ADL performance.

Differences in intervention composition and implementation conditions may partly explain these inconsistent findings. Previous studies have suggested that cognitive improvements may not sufficiently transfer to everyday performance when interventions do not include functional components [26]. Previous evidence also suggests that ADL improvements may not be observed when the intervention duration is insufficient [27]. In addition, differences in participants’ clinical stage have been suggested as a potential source of variation in outcomes [28,29,30], and the characteristics of the outcome measures used may also influence interpretation [28]. These factors should therefore inform the interpretation of the subgroup and meta-regression findings of the present review.

In the subgroup analyses, statistically significant pooled effects were observed within each subgroup, but no statistically significant between-subgroup differences were detected. Interventions that included functional components showed a tendency toward larger effect sizes, and studies using passive controls also showed larger effect sizes than those using active controls. The pooled effect sizes were also similar between DCR combined with another intervention and DCR alone. These patterns may be clinically and methodologically relevant because functional components may enhance the transfer of cognitive training to everyday activities, and passive controls may create a larger contrast with the intervention condition. However, because the number of studies within each subgroup was small, these subgroup findings should be interpreted as exploratory and inconclusive. The nonsignificant between-subgroup differences should not be interpreted as evidence that functional components, combined intervention status, or comparison type had no effect on ADL outcomes. Rather, the available evidence was insufficient to draw firm conclusions about whether these intervention or study characteristics moderate the effects of DCR on ADL performance.

The meta-regression analyses showed no statistically significant associations between ADL outcomes and dosage-related variables, including session length, weekly frequency, intervention duration, total number of sessions, and total training hours. A previous meta-analysis suggested that short intervention duration and low training intensity may partly explain the absence of ADL improvement [27]. However, the present meta-regression analyses were based on only 12 studies, and five continuous predictors were tested independently. Therefore, statistical power was limited, and false-negative findings cannot be ruled out. The nonsignificant findings should be interpreted as exploratory and inconclusive rather than as evidence that dosage-related characteristics had no effect on ADL outcomes. Future studies should examine how specific intervention components and dosage characteristics contribute to ADL improvement.

In addition, DCR was associated with a significant improvement in IADL performance. However, only four studies contributed to this finding. Few previous meta-analyses have examined IADL separately, and some have interpreted IADL together with ADL outcomes [28]. The present finding suggests that DCR may also have beneficial effects on more complex domains of everyday performance. A sensitivity analysis excluding the study that reported the IADL outcome using the Luton Index showed that the pooled IADL effect remained statistically significant. Nonetheless, the available evidence is limited and should be approached with caution. Due to the limited number of studies that reported on IADL outcomes, subgroup analysis and meta-regression were not conducted. Publication bias was also not assessed because the results would be unstable with a small number of studies [33,69].

Sensitivity analysis for ADL outcomes showed that the overall effect size did not change substantially when individual studies were sequentially removed. This suggests that the pooled ADL estimate was not driven by any single study. In the analysis of publication bias for the ADL outcome, visual examination of the funnel plot indicated slight asymmetry, whereas Egger’s test did not show statistical significance. However, a nonsignificant Egger’s test should not be interpreted as confirming the absence of publication bias, particularly given the limited number of studies included in the ADL meta-analysis. Therefore, the ADL findings should be interpreted with appropriate caution.

The methodological quality of the 18 included studies varied, with PEDro scores ranging from 4 to 8. None of the studies included in the review implemented blinding for either the subjects or the therapists. This limitation is closely related to the inherent characteristics of rehabilitation intervention studies, in which participant and therapist blinding is often difficult to achieve [70]. When blinding of participants and therapists is not feasible, assessor blinding can serve as a practical strategy to enhance the objectivity of outcome measurement in rehabilitation research [71]. In this review, assessor blinding was implemented in 11 studies. However, allocation concealment was insufficiently reported in a substantial proportion of studies. This issue should be considered when interpreting the findings because inadequate allocation concealment may lead to overestimation of treatment effects [72].

In the RoB 2 assessment, most included studies received an overall judgment of “some concerns,” whereas one study was judged to be at “high risk.” Domain-level uncertainty was most frequent for the randomization process and selection of the reported result, with approximately 72% of studies receiving “some concerns” judgments in each domain. These findings indicate that, although all included studies were RCTs, methodological uncertainty remained in several domains. In particular, risk of bias related to outcome reporting may be associated with the possibility of selective reporting and therefore warrants caution when interpreting intervention effects [37]. Accordingly, although the findings of this review showed a generally consistent direction of effect, they should be interpreted with consideration of the risk of bias in the included studies.

GRADE was applied only to the ADL and IADL outcomes included in the meta-analysis. For ADL, the certainty of evidence was judged to be moderate. This rating reflected a one-level downgrade for risk of bias, while no additional concerns were identified for inconsistency, indirectness, imprecision, or publication bias. Therefore, although the evidence for ADL outcomes can be interpreted with moderate confidence, further high-quality studies are needed to confirm the magnitude and generalizability of the effect. For IADL, the certainty of evidence was judged to be low. This rating reflected downgrading for risk of bias and imprecision, mainly because the analysis included relatively few studies and participants and had a relatively wide confidence interval. Accordingly, confidence in the evidence for IADL outcomes remains limited.

This review has several limitations. First, although functional cognition was prespecified as the primary outcome, no included study directly assessed functional cognition. As a result, ADL and IADL were analyzed as functional performance outcomes. This finding suggests that current DCR research has not sufficiently incorporated measures of functional cognition that reflect real-world task performance. Future studies should include performance-based measures that directly assess functional cognition to clarify how improvements in cognitive function translate into functional performance.

Second, evidence on IADL outcomes remains limited. Future high-quality studies should include IADL as a primary outcome to clarify whether DCR improves more complex domains of everyday performance.

Third, differences in the outcome measures used to assess functional performance may have influenced the interpretation of the findings. Although the included studies used standardized or validated instruments, the ADL meta-analysis combined observer-rated performance measures, such as the FIM, BI, MBI, and K-MBI, with the SIS-ADL, which reflects self-reported perceived difficulty in daily activities. Standardization using Hedges’ g allowed these outcomes to be synthesized on a common metric, but it did not eliminate conceptual differences among the outcome measures. In the leave-one-out sensitivity analysis, exclusion of Ho et al. [54], which used the SIS-ADL, did not materially change the pooled ADL effect. Nevertheless, measurement-related conceptual heterogeneity cannot be fully ruled out as a source of variation in the pooled estimates. Future studies should use more consistent and functionally relevant outcome measures.

Fourth, limitations in the methodological quality and risk of bias of the included studies may affect the interpretation and generalizability of the findings. In addition, most interventions were delivered in structured clinical settings under therapist supervision, whereas community-based and home-based applications were limited. Future large-scale RCTs with more rigorous study designs are needed. Further research should also examine the effects of DCR interventions that can be implemented in community-based and remote settings.

Finally, the review process had several limitations. Only English-language studies were included. Google Scholar screening was limited to the first 200 records for each search strategy, following methodological recommendations for managing Google Scholar searches in systematic reviews. These search-related limitations may have resulted in the omission of some potentially relevant studies. In addition, some studies could not be included in the meta-analysis because effect sizes could not be calculated from the available data, which may have restricted the scope of the meta-analysis. These limitations should be considered when interpreting the overall findings of this review.

## 5. Conclusions

This review suggests that DCR may contribute to improvements in ADL performance in individuals after stroke. A significant improvement was also observed for IADL performance, although this result should be interpreted cautiously because only four studies contributed data and the certainty of evidence was low. In addition, the prediction intervals for both ADL and IADL included no effect, indicating that the consistency of these observed effects in future trials remains uncertain. Moreover, because most included studies assessed outcomes only immediately after treatment, the clinical importance and long-term durability of the observed effects remain unclear. No included study directly assessed functional cognition, indicating a lack of performance-based assessments that evaluate the use of cognition during real-life task performance in DCR research. Therefore, the findings should be interpreted as evidence on ADL and IADL functional performance outcomes, not as direct evidence of effects on functional cognition.

Subgroup and meta-regression analyses did not detect statistically significant differences according to intervention components or dosage-related variables. Given the limited number of studies available for these analyses, these findings should be regarded as exploratory and inconclusive rather than confirmatory. Future RCTs should include functional performance outcomes, such as ADL and IADL, together with performance-based measures that directly assess functional cognition. When sufficient evidence has accumulated on intervention components and intervention dosage, future systematic reviews and meta-analyses should re-examine how these factors influence the effects of DCR on functional performance. Such evidence may support DCR as an integrated intervention approach for improving functional performance and strengthen evidence-based practice in post-stroke cognitive rehabilitation.

## Figures and Tables

**Table 1:** Characteristics of the included studies.

Author (Year)	Country	Setting	Participants (N, Age)	Intervention	Duration	Comparison	Time Point	Outcomes Measure
Camara et al. (2025) [48]	Portugal	Hospital	EG1: *n* = 10, 54.5 ± 9.93 EG2: *n* = 10, 57.3 ± 13.55 CG: *n* = 10, 62.2 ± 10.47	EG1: Adaptive tablet-based cognitive training EG2: Adaptative paper-and-pencil cognitive training	6 weeks	Waiting list	Post (6 weeks) Follow-up (18 weeks)	IAFAI
Chen et al. (2024) [49]	China	Hospital	EG1: *n* = 18, 61.06 ± 3.08 EG2: *n* = 18, 62.44 ± 2.76 EG3: *n* = 18, 58.50 ± 3.75 CG: *n* = 18, 65.17 ± 3.26	EG1: CBCT + tDCS EG2: CBCT EG3: tDCS	3 weeks	CCT	Post (3 weeks)	Lawton IADL
Cho et al. (2016) [50]	Korea	Hospital	EG1: *n* = 14, 63.00 ± 5.40 EG2: *n* = 14, 62.20 ± 6.20 CG: *n* = 16, 64.00 ± 8.80	EG1: CBCT EG2: Neurofeedback(EEG)	6 weeks	Usual care	Post (6 weeks)	FIM
Faria et al. (2016) [51]	Portugal	Hospital	EG: *n* = 9, 58 (48–71) CG: *n* = 9, 53 (50.5–65.5)	VR-based ADL simulation cognitive rehabilitation	4~6 weeks	CCT	Post (4~6 weeks)	SIS-ADL
Feng et al. (2025) [52]	China	Hospital	EG: *n* = 30, 63.5 (IQR = 14) CG: *n* = 30, 64.5 (IQR = 15)	CBCT	4 weeks	Usual care	Post (4 weeks)	MBI
He et al. (2021) [53]	China	Hospital	EG: *n* = 32, 66.00 ± 6.90 CG: *n* = 32, 67.00 ± 5.90	Cognitive rehabilitation strategy module based on the eye movement technique	6 weeks	CCT	Post (6 weeks) Follow-up (10 weeks)	MBI
Ho et al. (2022) [54]	Taiwan	Hospital	EG: *n* = 19, 63.63 ± 11.27 CG: *n* = 20, 65.50 ± 8.28	CBCT	12 weeks	CCT	Post (12 weeks) Follow-up (16 weeks)	SIS-ADL
Li et al. (2021) [55]	China	Hospital	EG: *n* = 15, 62 (IQR = 24) CG: *n* = 15, 57 (IQR = 32)	Serious game–based cellphone AR system + Conventional OT	2 weeks	Conventional OT	Post (2 weeks)	BI
Liu et al. (2024) [56]	Taiwan	Hospital	EG1: *n* = 13, 53.10 ± 17.60 EG2: *n* = 13, 56.10 ± 11.00 CG: *n* = 13, 54.70 ± 9.60	EG1: CBCT + Sequential AE EG2: CBCT + Simultaneous AE	12 weeks	Unstructured cognitive activities	Post (12 weeks)	Lawton IADL
Liu et al. (2023) [57]	China	Hospital	EG: *n* = 15, 75.93 ± 6.81 CG: *n* = 15, 73.40 ± 7.50	Immersive VR–based puzzle game	6 weeks	CCT	Post (6 weeks)	MBI
Oliveri et al. (2023) [58]	Italy	Hospital	EG: *n* = 15, 65.10 ± 14.60 CG: *n* = 15, 53.90 ± 12.40	Digitized prism adaptation + Serious games cognitive training	2 weeks	CCT	Post (2 weeks)	BI
Park & Ha (2023) [59]	Korea	Hospital	EG: *n* = 20, 62.50 ± 4.70 CG1: *n* = 20, 62.00 ± 3.00 CG2: *n* = 20, 62.50 ± 4.80	Immersive VR-based cognitive training + workbook	8 weeks	CG1: CBCT CG2: CCT	Mid (4 weeks) Post (8 weeks)	K-MBI
Soni et al. (2025) [60]	India	Home-based	EG: *n* = 25, 51.08 ± 6.25 CG: *n* = 25, 49.40 ± 6.38	CBCT	4 weeks	CCT	Post (4 weeks)	BI, Lawton IADL
Veisi-Pirkoohi et al. (2020) [61]	Iran	Rehabilitation clinic	EG: *n* = 25, 52.92 ± 10.44 CG: *n* = 25, 58.80 ± 13.32	CBCT	5 weeks	No intervention	Post (5 weeks)	BI, Luton Index
Yeh et al. (2022) [62]	Taiwan	Hospital	EG1: *n* = 20, 53.05 ± 14.53 EG2: *n* = 18, 60.17 ± 12.13 CG: *n* = 18, 57.36 ± 12.17	EG1: CBCT + Sequential AE EG2: CBCT	12 weeks	AE	Post (12 weeks)	FIM, Lawton IADL
Yoo et al. (2015) [63]	Korea	Hospital	EG: *n* = 23, 53.20 ± 8.80 CG: *n* = 23, 56.30 ± 7.90	CBCT	5 weeks	Usual care	Post (5 weeks)	FIM
He et al. (2021) [64]	China	Hospital	EG1: *n* = 23, 57 (51–65) EG2: *n* = 24, 57 (48.25–64) CG: *n* = 25, 58 (51.5–66)	EG1: CBCT + BADL training(video) EG2: CBCT + BADL training(demonstration)	3 weeks	CCT + BADL training(demonstration)	Post (3 weeks)	MBI, Lawton IADL
Zhang et al. (2025) [65]	China	Hospital	EG: *n* = 24, 55.96 ± 11.67 CG: *n* = 24, 58.29 ± 13.47	Eye-tracking technology-based VST + Conventional USN training	4 weeks	Conventional USN training	Post (4 weeks)	MBI

Age is reported in years as mean ± SD, median (Q1–Q3), or median (IQR). ADL: activities of daily living; AE: aerobic exercise; AR: augmented reality; BADL: basic activities of daily living; BI: Barthel Index; CBCT: computer-based cognitive training; CCT: conventional cognitive training; CG: control group; EEG: electroencephalography; EG: experimental group; FIM: Functional Independence Measure; IAFAI: Adults and Older Adults Functional Assessment Inventory; IADL: instrumental activities of daily living; IQR: interquartile range; M: median; MBI: Modified Barthel Index; OT: occupational therapy; SD: standard deviation; SIS: Stroke Impact Scale; tDCS: transcranial direct current stimulation; USN: unilateral spatial neglect; VR: virtual reality; VST: visual scanning training.

**Table 2:** Characteristics of the interventions.

Author (Year)	Intervention	Technology Approach	Device	Combined Intervention	Functional Component	Delivery Mode	Session Length (min)	Frequency (Session/Week)	Duration (Weeks)
Camara et al. (2025) [48]	Adaptive tablet-based cognitive training (NeuroAIreh@b)	Mobile app	Tablet	No	Yes	Therapist supervised	30	2	6
Chen et al. (2024) [49]	CBCT (66nao Brain rehabilitation system)	Computer-based	Tablet	tDCS (Simultaneous)	No	Therapist supervised	20	5	3
Chen et al. (2024) [49]	CBCT (66nao Brain rehabilitation system)	Computer-based	Tablet	No	No	Therapist supervised	20	5	3
Cho et al. (2016) [50]	CBCT (Rehacom)	Computer-based	PC	No	No	Therapist supervised	30	5	6
Faria et al. (2016) [51]	VR-based ADL simulation cognitive rehabilitation (Reh@City)	VR (Non immersive)	PC Joystick	No	Yes	Therapist supervised	20	Not reported	4~6
Feng et al. (2025) [52]	CBCT (Flex table digital OT assessment and training)	Computer-based	PC	No	Yes	Therapist supervised	20	6	4
He et al. (2021) [53]	Eye movement–based cognitive training system (JZ-RZ-1020)	Computer-based	PC	No	No	Therapist supervised	20	6	6
Ho et al. (2022) [54]	CBCT (Lumosity)	Computer-based	PC	No	No	Therapist supervised	20	2	12
Li et al. (2021) [55]	Serious game-based CARS	AR	Cellphone	Conventional OT	No	Therapist supervised	60	5	2
Liu et al. (2024) [56]	CBCT (BrainHQ)	Computer-based	PC	AE (Sequential)	No	Therapist supervised	60	3	12
Liu et al. (2024) [56]	CBCT (BrainHQ)	Computer-based	PC	AE (Simultaneous)	No	Therapist supervised	60	3	12
Liu et al. (2023) [57]	Immersive VR–based puzzle game	VR (Immersive)	Tablet HMD	No	Yes	Therapist supervised	15	6	6
Oliveri et al. (2023) [58]	Serious games cognitive training	Computer-based	Tablet	Prism adaptation (Sequential)	Yes	Therapist supervised	40	Not reported	2
Park & Ha (2023) [59]	VR based cognitive training	VR (Immersive)	Goggles/ controllers	Task related to VR contents using workbook	No	Therapist supervised	30	5	8
Park & Ha (2023) [59]	CBCT (ComCog)	Computer-based	PC	No	No	Therapist supervised	30	5	8
Soni et al. (2025) [60]	CBCT	Computer-based	PC/laptop/tablet/mobile phone	No	No	Remotely delivered, weekly therapist review	60	5	4
Veisi-Pirkoohi et al. (2020) [61]	CBCT (Rehacom)	Computer-based	PC	No	No	Therapist supervised	45	2	5
Yeh et al. (2022) [62]	CBCT (BrainHQ)	Computer-based	PC	AE (Sequential)	No	Therapist supervised	60	3	12
Yeh et al. (2022) [62]	CBCT (BrainHQ)	Computer-based	PC	No	No	Therapist supervised	60	3	12
Yoo et al. (2015) [63]	CBCT (Rehacom)	Computer-based	PC	No	No	Therapist supervised	30	5	5
He et al. (2021) [64]	CBCT	Computer-based	PC	Video-based self regulation learning (BADL training)	Yes	Therapist supervised	90	5	3
He et al. (2021) [64]	CBCT	Computer-based	PC	Demonstration learning (BADL training)	Yes	Therapist supervised	90	5	3
Zhang et al. (2025) [65]	Eye-tracking technology-based VST	Computer-based	EMT instrument	Conventional USN training	No	Therapist supervised	30	5	4

AE: aerobic exercise; BADL: basic activities of daily living; CARS: cellphone augmented reality system; CBCT: computer-based cognitive training; EMT: eye tracking training; HMD: head mounted display; OT: occupational therapy; tDCS: transcranial direct current stimulation; VR: virtual reality; VST: visual scanning training.

**Table 3:** Methodological quality of the included studies.

Study	1	2	3	4	5	6	7	8	9	10	11	Total Score
Camara et al. [48]	1	1	0	1	0	0	0	0	0	1	1	4/10
Chen et al. [49]	1	1	1	1	0	0	1	1	1	1	1	8/10
Cho et al. [50]	1	1	0	1	0	0	0	0	1	1	1	5/10
Faria et al. [51]	1	1	0	1	0	0	0	1	1	1	1	6/10
Feng et al. [52]	1	1	0	1	0	0	1	1	1	1	1	7/10
He et al. [53]	1	1	1	1	0	0	1	1	1	1	1	8/10
Ho et al. [54]	1	1	1	1	0	0	0	1	0	1	1	6/10
Li et al. [55]	1	1	1	1	0	0	1	1	1	1	1	8/10
Liu et al. [56]	1	1	1	1	0	0	1	0	0	1	0	5/10
Liu et al. [57]	1	1	0	1	0	0	0	1	1	1	0	5/10
Oliveri et al. [58]	1	1	0	1	0	0	1	1	1	1	1	7/10
Park & Ha [59]	1	1	0	1	0	0	1	1	0	1	1	6/10
Soni et al. [60]	1	1	0	1	0	0	1	0	0	1	1	5/10
Veisi-Pirkoohi et al. [61]	1	1	0	0	0	0	0	1	1	1	1	5/10
Yeh et al. [62]	1	1	0	1	0	0	1	1	1	1	1	7/10
Yoo et al. [63]	0	1	0	1	0	0	0	1	1	0	1	5/10
He et al. [64]	1	1	0	0	0	0	1	1	0	1	1	5/10
Zhang et al. [65]	1	1	0	1	0	0	1	1	1	1	1	7/10

Items: 1: eligibility criteria; 2: random allocation; 3: allocation concealment; 4: baseline comparability; 5: blinding of subjects; 6: blinding of therapists; 7: blinding of assessors; 8: adequate follow-up; 9: intention-to-treat analysis; 10: between-group comparisons; 11: point estimates and variability; yes = 1; no = 0.

**Table 4:** Subgroup analysis of ADL outcomes.

Variable	Category	k	Hedges’ g	95% CI	*p*-Value	Q (df) (Between)	*p*-Value (Between)
Functional component	Yes	4	0.697	0.334–1.060	<0.001	2.344 (1)	0.126
	No	8	0.353	0.102–0.604	0.006		
Combined intervention status	Yes ^a^	5	0.455	0.093–0.816	0.014	0.002 (1)	0.963
	No ^b^	7	0.466	0.163–0.770	0.003		
Comparison type	Active	8	0.376	0.106–0.646	0.006	1.142 (1)	0.285
	Passive	4	0.626	0.256–0.996	0.001		

^a^ DCR combined with another intervention. ^b^ DCR alone, also referred to as stand-alone DCR.

**Table 5:** Meta-regression analysis of ADL outcomes.

Variable	β	SE	95% CI	*p*-Value
Session length (min)	0.001	0.006	−0.011–0.012	0.933
Weekly frequency	0.031	0.094	−0.153–0.215	0.740
Intervention duration (weeks)	−0.009	0.039	−0.086–0.068	0.814
Total number of sessions	0.001	0.013	−0.024–0.026	0.921
Total training hours	0.001	0.014	−0.027–0.029	0.960

**Table 6:** GRADE certainty of evidence for ADL and IADL outcomes.

Outcome	Studies (RCTs)	Participants	Hedges’ g (95% CI)	Certainty
ADL	12	503	0.463 (95% CI = 0.241–0.684)	Moderate ^a^
IADL	4	186	0.580 (95% CI = 0.248–0.912)	Low ^a,b^

^a^ Downgraded one level for risk of bias because all included studies were rated as having some concerns in the overall RoB 2 assessment. ^b^ Downgraded one level for imprecision due to the small number of studies and participants and the relatively wide confidence interval.

## Data Availability

No new primary data were collected in this study. Data were extracted from the published studies included in the analysis, and the summarized data are presented within this article.

## Supplementary Materials

The following supporting information can be downloaded at https://www.mdpi.com/article/10.3390/healthcare14152355/s1. Table S1: Search strategies used for each database; Table S2: Cognitive impairment criteria and baseline participant characteristics of the included studies; Table S3: Summary of studies retained in the narrative synthesis but excluded from the meta-analysis; Figure S1: Sensitivity analysis for the IADL meta-analysis excluding the study that used the Luton Index.

## Abbreviations

The following abbreviations are used in this manuscript:

ADL	Activities of daily living
AR	Augmented reality
BI	Barthel Index
CBCT	Computer-based cognitive training
CI	Confidence interval
CMA	Comprehensive Meta-Analysis
DCR	Digital cognitive rehabilitation
EFPT	Executive Function Performance Test
FIM	Functional Independence Measure
GRADE	Grading of Recommendations Assessment, Development and Evaluation
IAFAI	Adults and Older Adults Functional Assessment Inventory
IADL	Instrumental activities of daily living
K-MBI	Korean Modified Barthel Index
K-MMSE-2	Korean Mini-Mental State Examination, second edition
MBI	Modified Barthel Index
MMSE	Mini-Mental State Examination
MoCA	Montreal Cognitive Assessment
PEDro	Physiotherapy Evidence Database
PRISMA	Preferred Reporting Items for Systematic Reviews and Meta-Analyses
PSCI	Post-stroke cognitive impairment
RCTs	Randomized controlled trials
RoB 2	Risk of Bias 2
SIS	Stroke Impact Scale
SMD	Standardized mean difference
tDCS	Transcranial direct current stimulation
VR	Virtual reality
VCI	Vascular cognitive impairment
WCPA	Weekly Calendar Planning Activity
