# Supplementary material for: Effects of Digital Cognitive Rehabilitation on Functional Performance After Stroke: A Systematic Review and Meta-Analysis of ADL and IADL Outcomes
Source: Healthcare (Basel). 2026 Aug 2;14(15):2355. doi: 10.3390/healthcare14152355 (PMC13465463; doi:10.3390/healthcare14152355)
Supplement: Supplementary file 1 [file healthcare-14-02355-s001.zip › Figure S1. Sensitivity analysis for the IADL meta-analysis excluding the study that used the Luton Index.pdf]

Study name	Comparison	Outcome	Statistics with study removed						
			Point	Standard error	Variance	Lower limit	Upper limit	Z-Value	p-Value
Soni et al.(2025)	active	Lawton IADL	0.681	0.200	0.040	0.289	1.073	3.401	0.001
Veisi-Pirkoohi et al.(2020)	passive	Luton Index	0.502	0.215	0.046	0.080	0.924	2.329	0.020
Yeh et al.(2022)	acitve	Lawton IADL	0.672	0.192	0.037	0.296	1.048	3.501	0.000
He et al.(2021)	active	Lawton IADL	0.467	0.175	0.030	0.124	0.809	2.673	0.008
Pooled			0.580	0.170	0.029	0.248	0.912	3.422	0.001
Prediction Interval			0.580	0.000	0.000	-0.436	1.596	0.000	0.000

Hedges's g (95% CI) with study removed

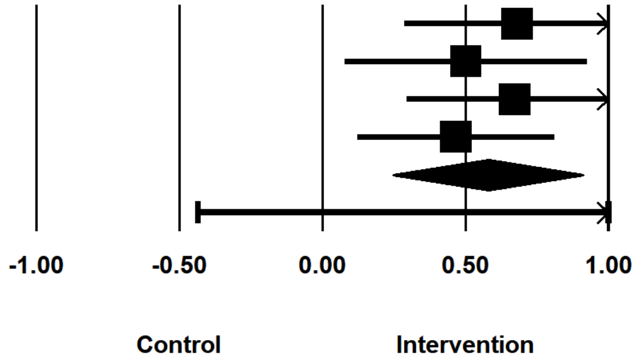

Supplementary Figure S1. Sensitivity analysis for the IADL meta-analysis excluding the study that used the Luton Index.
