# Supplementary material for: Effects of Digital Cognitive Rehabilitation on Functional Performance After Stroke: A Systematic Review and Meta-Analysis of ADL and IADL Outcomes
Source: Healthcare (Basel). 2026 Aug 2;14(15):2355. doi: 10.3390/healthcare14152355 (PMC13465463; doi:10.3390/healthcare14152355)
Supplement: Supplementary file 1 [file healthcare-14-02355-s001.zip › Table S1. Search strategies used for each database.pdf]

Supplementary Table S1. Search strategies used for each database.

Database	Search line	Search strategy	Records identified
PubMed	Search 1	(stroke[MeSH Terms] OR post-stroke[Title/Abstract] OR "cerebrovascular accident"[Title/Abstract] OR "stroke survivor*"[Title/Abstract] OR "stroke patient*"[Title/Abstract])<br>AND ("digital"[Title/Abstract] OR "digital cognitive rehabilitation"[Title/Abstract] OR "virtual reality"[Title/Abstract] OR "serious game*"[Title/Abstract] OR "telerehabilitation"[Title/Abstract] OR "mobile"[Title/Abstract] OR "mobile app*"[Title/Abstract] OR "computer"[Title/Abstract] OR "computer-based training"[Title/Abstract] OR "computer assist*"[Title/Abstract] OR "technology-based rehabilitation"[Title/Abstract])<br>AND ("functional cognition"[Title/Abstract] OR "performance-based cognition"[Title/Abstract] OR "occupational performance"[Title/Abstract] OR "functional performance"[Title/Abstract])<br>AND (randomized controlled trial[Publication Type] OR randomized[Title/Abstract] OR randomised[Title/Abstract])<br>AND ("2010/01/01"[Date - Publication] : "2025/12/30"[Date - Publication]) AND (english[Language])	5
PubMed	Search 2	(stroke[MeSH Terms] OR post-stroke[tiab] OR "cerebrovascular accident"[tiab] OR "stroke survivor*"[tiab] OR "stroke patient*"[tiab]) AND ("digital" OR "digital cognitive rehabilitation" OR "virtual reality" OR "serious game*" OR "telerehabilitation" OR "mobile app*" OR "computer-based training" OR "technology-based rehabilitation") AND ("executive function performance test" OR EFPT OR "weekly calendar planning activity" OR WCPA OR "kitchen task assessment" OR KTA OR "cognitive performance test" OR CPT OR "performance assessment of self-care skills" OR PASS OR "multiple errands test" OR MET OR "kettle test" OR "complex task performance assessment" OR CTPA OR "functional cognitive assessment scale" OR FUCAS OR "observed tasks of daily living" OR OTDL OR "multiple object test" OR "naturalistic action test" OR "pillbox test" OR "medication management test" OR "managing finances" OR "shopping task")<br>AND (randomized controlled trial[pt] OR randomized[tiab] OR randomised[tiab])<br>AND ("2010/01/01"[Date - Publication] : "2025/12/30"[Date - Publication]) AND (english[Language])	13
PubMed	Search 3	(stroke[MeSH Terms] OR post-stroke[Title/Abstract] OR "cerebrovascular accident"[Title/Abstract] OR "stroke survivor*"[Title/Abstract] OR "stroke patient*"[Title/Abstract])<br>AND ("digital"[Title/Abstract] OR "digital cognitive rehabilitation"[Title/Abstract] OR "virtual reality"[Title/Abstract] OR "serious game*"[Title/Abstract] OR "telerehabilitation"[Title/Abstract] OR "mobile"[Title/Abstract] OR "mobile app*"[Title/Abstract] OR "computer"[Title/Abstract] OR "computer-based training"[Title/Abstract] OR "computer assist*"[Title/Abstract] OR "technology-based rehabilitation"[Title/Abstract])<br>AND ("activities of daily living"[Title/Abstract] OR ADL[Title/Abstract] OR "instrumental activities of daily living"[Title/Abstract] OR IADL[Title/Abstract] OR "cognitive function"[Title/Abstract] OR cognition[Title/Abstract])<br>AND (randomized controlled trial[Publication Type] OR randomized[Title/Abstract] OR randomised[Title/Abstract])<br>AND ("2010/01/01"[Date - Publication] : "2025/12/30"[Date - Publication]) AND (english[Language])	76
		Total	94

Supplementary Table S1. *Cont.*

Database	Search line	Search strategy	Records identified
Embase	Search 1	(stroke/exp OR stroke:ti,ab OR poststroke:ti,ab OR "cerebrovascular accident":ti,ab OR "stroke survivor*":ti,ab OR "stroke patient*":ti,ab) AND ("digital":ti,ab OR "digital cognitive rehabilitation":ti,ab OR "virtual reality":ti,ab OR "serious game*":ti,ab OR telerehabilitation:ti,ab OR mobile:ti,ab OR "mobile app*":ti,ab OR computer:ti,ab OR "computer-based training":ti,ab OR "computer assist*":ti,ab OR "technology-based rehabilitation":ti,ab) AND ("functional cognition":ti,ab OR "performance-based cognition":ti,ab OR "occupational performance":ti,ab OR "functional performance":ti,ab) AND (randomized controlled trial/exp OR random*:ti,ab) AND [english]/lim AND [humans]/lim AND [2010-2025]/py	27
	Search 2	(stroke/exp OR stroke:ti,ab OR poststroke:ti,ab OR "cerebrovascular accident":ti,ab OR "stroke survivor*":ti,ab OR "stroke patient*":ti,ab) AND ("digital":ti,ab OR "digital cognitive rehabilitation":ti,ab OR "virtual reality":ti,ab OR "serious game*":ti,ab OR telerehabilitation:ti,ab OR mobile:ti,ab OR "mobile app*":ti,ab OR computer:ti,ab OR "computer-based training":ti,ab OR "computer assist*":ti,ab OR "technology-based rehabilitation":ti,ab) AND ("executive function performance test":ti,ab OR EFPT:ti,ab OR "weekly calendar planning activity":ti,ab OR WCPA:ti,ab OR "kitchen task assessment":ti,ab OR KTA:ti,ab OR "cognitive performance test":ti,ab OR CPT:ti,ab OR "performance assessment of self-care skills":ti,ab OR PASS:ti,ab OR "multiple errands test":ti,ab OR MET:ti,ab OR "kettle test":ti,ab OR "complex task performance assessment":ti,ab OR CTPA:ti,ab OR "functional cognitive assessment scale":ti,ab OR FUCAS:ti,ab OR "observed tasks of daily living":ti,ab OR OTDL:ti,ab OR "multiple object test":ti,ab OR "naturalistic action test":ti,ab OR "pillbox test":ti,ab OR "medication management test":ti,ab OR "managing finances":ti,ab OR "shopping task":ti,ab) AND (randomized controlled trial/exp OR random*:ti,ab) AND [english]/lim AND [humans]/lim AND [2010-2025]/py	127
	Search 3	(stroke/exp OR stroke:ti,ab OR poststroke:ti,ab OR "cerebrovascular accident":ti,ab OR "stroke survivor*":ti,ab OR "stroke patient*":ti,ab) AND ("digital":ti,ab OR "digital cognitive rehabilitation":ti,ab OR "virtual reality":ti,ab OR "serious game*":ti,ab OR telerehabilitation:ti,ab OR mobile:ti,ab OR "mobile app*":ti,ab OR computer:ti,ab OR "computer-based training":ti,ab OR "computer assist*":ti,ab OR "technology-based rehabilitation":ti,ab) AND ("activities of daily living":ti,ab OR ADL:ti,ab OR "instrumental activities of daily living":ti,ab OR IADL:ti,ab OR "cognitive function":ti,ab OR cognition:ti,ab) AND (randomized controlled trial/exp OR random*:ti,ab) AND [english]/lim AND [humans]/lim AND [2010-2025]/py	293
		Total	447

Supplementary Table S1. *Cont.*

Database	Search line	Search strategy	Records identified
CINAHL Complete	#1	TI(stroke OR "post-stroke" OR "cerebrovascular accident" OR "stroke survivor*") OR AB(stroke OR "post-stroke" OR "cerebrovascular accident" OR "stroke survivor*")	90,786
	#2	TI(digital OR "digital cognitive rehabilitation" OR "virtual reality" OR "serious game*" OR telerehabilitation OR "tele-rehabilitation" OR mobile OR "mobile app*" OR computer OR "computer-based" OR "computer assisted" OR "technology-based" OR "technology assisted") OR AB(digital OR "digital cognitive rehabilitation" OR "virtual reality" OR "serious game*" OR telerehabilitation OR "tele-rehabilitation" OR mobile OR "mobile app*" OR computer OR "computer-based" OR "computer assisted" OR "technology-based" OR "technology assisted")	112,790
	#3	TI("functional cognition" OR "performance-based cognition" OR "functional performance" OR "occupational performance") OR AB("functional cognition" OR "performance-based cognition" OR "functional performance" OR "occupational performance")	4,243
	#4	TI("executive function performance test" OR EFPT OR "weekly calendar planning activity" OR WCPA OR "kitchen task assessment" OR KTA OR "cognitive performance test" OR CPT OR "performance assessment of self-care skills" OR PASS OR "multiple errands test" OR MET OR "big-store multiple errands test" OR "kettle test" OR CTPA OR "complex task performance assessment" OR FUCAS OR OTDL OR "observed tasks of daily living") OR AB("executive function performance test" OR EFPT OR "weekly calendar planning activity" OR WCPA OR "kitchen task assessment" OR KTA OR "cognitive performance test" OR CPT OR "performance assessment of self-care skills" OR PASS OR "multiple errands test" OR MET OR "big-store multiple errands test" OR "kettle test" OR CTPA OR "complex task performance assessment" OR FUCAS OR OTDL OR "observed tasks of daily living") OR TI("multiple object test" OR "naturalistic action test" OR "pillbox test" OR "medication management test" OR "managing finances" OR "shopping task" OR "bill paying" OR "checkbook balancing" OR "telephone use test" OR "front desk duty" OR "charge of quarters" OR "day-out task" OR "night out task" OR "day-to-day task*" OR "day to day task*" OR multitasking OR "multitasking test" OR "multitasking assessment") OR AB("multiple object test" OR "naturalistic action test" OR "pillbox test" OR "medication management test" OR "managing finances" OR "shopping task" OR "bill paying" OR "checkbook balancing" OR "telephone use test" OR "front desk duty" OR "charge of quarters" OR "day-out task" OR "night out task" OR "day-to-day task*" OR "day to day task*" OR multitasking OR "multitasking test" OR "multitasking assessment")	96,862
	#5	TI("activities of daily living" OR ADL OR "instrumental activities of daily living" OR IADL OR "cognitive function" OR cognition) OR AB("activities of daily living" OR ADL OR "instrumental activities of daily living" OR IADL OR "cognitive function" OR cognition)	56,100
	#6	TI("randomized controlled trial" OR randomized OR randomised OR RCT) OR AB("randomized controlled trial" OR randomized OR randomised OR RCT)	279,803
	Search 1	#1 AND #2 AND #3 AND #6	4
	Search 2	#1 AND #2 AND #4 AND #6	31
	Search 3	#1 AND #2 AND #5 AND #6	99
		Total	134

Supplementary Table S1. *Cont.*

Database	Search line	Search strategy	Records identified
PsycArticles	#1	TI(stroke OR "post-stroke" OR "cerebrovascular accident" OR "stroke survivor*") OR AB(stroke OR "post-stroke" OR "cerebrovascular accident" OR "stroke survivor*")	197
	#2	TI(digital OR "digital cognitive rehabilitation" OR "virtual reality" OR "serious game*" OR telerehabilitation OR "tele-rehabilitation" OR mobile OR "mobile app*" OR computer OR "computer-based" OR "computer assisted" OR "technology-based" OR "technology assisted") OR AB(digital OR "digital cognitive rehabilitation" OR "virtual reality" OR "serious game*" OR telerehabilitation OR "tele-rehabilitation" OR mobile OR "mobile app*" OR computer OR "computer-based" OR "computer assisted" OR "technology-based" OR "technology assisted")	2,965
	#3	TI("functional cognition" OR "performance-based cognition" OR "functional performance" OR "occupational performance") OR AB("functional cognition" OR "performance-based cognition" OR "functional performance" OR "occupational performance")	21
	#4	TI("executive function performance test" OR EFPT OR "weekly calendar planning activity" OR WCPA OR "kitchen task assessment" OR KTA OR "cognitive performance test" OR CPT OR "performance assessment of self-care skills" OR PASS OR "multiple errands test" OR MET OR "big-store multiple errands test" OR "kettle test" OR CTPA OR "complex task performance assessment" OR FUCAS OR OTDL OR "observed tasks of daily living") OR AB("executive function performance test" OR EFPT OR "weekly calendar planning activity" OR WCPA OR "kitchen task assessment" OR KTA OR "cognitive performance test" OR CPT OR "performance assessment of self-care skills" OR PASS OR "multiple errands test" OR MET OR "big-store multiple errands test" OR "kettle test" OR CTPA OR "complex task performance assessment" OR FUCAS OR OTDL OR "observed tasks of daily living") OR TI("multiple object test" OR "naturalistic action test" OR "pillbox test" OR "medication management test" OR "managing finances" OR "shopping task" OR "bill paying" OR "checkbook balancing" OR "telephone use test" OR "front desk duty" OR "charge of quarters" OR "day-out task" OR "night out task" OR "day-to-day task*" OR "day to day task*" OR multitasking OR "multitasking test" OR "multitasking assessment") OR AB("multiple object test" OR "naturalistic action test" OR "pillbox test" OR "medication management test" OR "managing finances" OR "shopping task" OR "bill paying" OR "checkbook balancing" OR "telephone use test" OR "front desk duty" OR "charge of quarters" OR "day-out task" OR "night out task" OR "day-to-day task*" OR "day to day task*" OR multitasking OR "multitasking test" OR "multitasking assessment")	1,660
	#5	TI("activities of daily living" OR ADL OR "instrumental activities of daily living" OR IADL OR "cognitive function" OR cognition) OR AB("activities of daily living" OR ADL OR "instrumental activities of daily living" OR IADL OR "cognitive function" OR cognition)	3,463
	#6	TI("randomized controlled trial" OR randomized OR randomised OR RCT) OR AB("randomized controlled trial" OR randomized OR randomised OR RCT)	3,374
	Search 1	#1 AND #2 AND #3 AND #6	0
	Search 2	#1 AND #2 AND #4 AND #6	1
	Search 3	#1 AND #2 AND #5 AND #6	1
		Total	2

Supplementary Table S1. *Cont.*

Database	Search line	Search strategy	Records identified
Cochrane Library	Search 1	(stroke OR "post-stroke" OR "cerebrovascular accident" OR "stroke survivor*") AND (digital OR "digital cognitive rehabilitation" OR "virtual reality" OR (serious NEXT game*) OR telerehabilitation OR "tele-rehabilitation" OR mobile OR (mobile NEXT app*) OR computer OR (computer NEXT based) OR (computer NEXT assisted) OR (technology NEXT based) OR (technology NEXT assisted)) AND ("functional cognition" OR "performance-based cognition" OR "functional performance" OR "occupational performance")	130
	Search 2	(stroke OR "post-stroke" OR "cerebrovascular accident" OR "stroke survivor*") AND (digital OR "digital cognitive rehabilitation" OR "virtual reality" OR (serious NEXT game*) OR telerehabilitation OR "tele-rehabilitation" OR mobile OR (mobile NEXT app*) OR computer OR (computer NEXT based) OR (computer NEXT assisted) OR (technology NEXT based) OR (technology NEXT assisted)) AND ("executive function performance test" OR EFPT OR "weekly calendar planning activity" OR WCPA OR "kitchen task assessment" OR KTA OR "cognitive performance test" OR CPT OR "performance assessment of self-care skills" OR PASS OR "multiple errands test" OR MET OR "big-store multiple errands test" OR "kettle test" OR CTPA OR "complex task performance assessment" OR FUCAS OR OTDL OR "observed tasks of daily living" OR "multiple object test" OR "naturalistic action test" OR "pillbox test" OR "medication management test" OR "managing finances" OR "shopping task" OR "bill paying" OR "checkbook balancing" OR "telephone use test" OR "front desk duty" OR "charge of quarters" OR "day-out task" OR "night out task" OR (day NEXT to NEXT day NEXT task*) OR multitasking OR "multitasking test")	421
	Search 3	(stroke OR "post-stroke" OR "cerebrovascular accident" OR "stroke survivor*") AND (digital OR "digital cognitive rehabilitation" OR "virtual reality" OR (serious NEXT game*) OR telerehabilitation OR "tele-rehabilitation" OR mobile OR (mobile NEXT app*) OR computer OR (computer NEXT based) OR (computer NEXT assisted) OR (technology NEXT based) OR (technology NEXT assisted)) AND ("activities of daily living" OR ADL OR "instrumental activities of daily living" OR IADL OR "cognitive function" OR cognition)	2003
		Total	2,554

Supplementary Table S1. *Cont.*

Database	Search line	Search strategy	Records identified
Google Scholar	Search 1	stroke AND ("digital cognitive rehabilitation" OR "computer-based cognitive training" OR "technology-assisted cognitive training")	200
	Search 2	stroke AND ("virtual reality" OR "serious game" OR "mobile app" OR telerehabilitation) AND (cognitive OR cognition)	200
	Search 3	stroke AND ("cognitive training" OR "cognitive rehabilitation") AND ("ADL" OR "IADL" OR "activities of daily living")	200
		Total	600

Note. For Google Scholar, the first 200 records from each search strategy were screened because Google Scholar displays results in order of relevance and does not provide database-style controlled indexing.
