# Supplementary material for: Effects of Digital Cognitive Rehabilitation on Functional Performance After Stroke: A Systematic Review and Meta-Analysis of ADL and IADL Outcomes
Source: Healthcare (Basel). 2026 Aug 2;14(15):2355. doi: 10.3390/healthcare14152355 (PMC13465463; doi:10.3390/healthcare14152355)
Supplement: Supplementary file 1 [file healthcare-14-02355-s001.zip › Table S2. Cognitive impairment criteria and baseline participant characteristics of the included studies.pdf]

Supplementary Table S2. Cognitive impairment criteria and baseline participant characteristics of the included studies.

Author(year)	Cognitive impairment criteria or diagnosis	Stroke type	Time since stroke	Stroke severity	Baseline cognitive status	Baseline functional status
Camara et al. (2025) [48]	MoCA score (≥ 1.5 SD below the normative mean)	EG1: H (6), I (4)<br>EG2: H (5), I (5)<br>CG: H (2), I (8)	EG1: 19.70 ± 25.45 mo<br>EG2: 23.50 ± 19.15 mo<br>CG: 15.20 ± 12.54 mo	Not reported	MoCA<br>EG1: 19.50 (IQR=6.50)<br>EG2: 20.00 (IQR=6.50)<br>CG: 21.00 (IQR=3.25)	IAFAI<br>EG1: 25.36 (IQR=23.74)<br>EG2: 42.36 (IQR=29.80)<br>CG: 31.73 (IQR=28.93)
Chen et al. (2024) [49]	MoCA score < 26	Ischaemic	EG1: 22.5 (16.5-22.5) d<br>EG2: 38.5 (12.5-99.3) d<br>EG3: 31.0 (24.5-74.5) d<br>CG: 29.5 (15.3-95.8) d	Not reported	MoCA<br>EG1: 14.78 ± 4.93<br>EG2: 14.89 ± 5.83<br>EG3: 12.72 ± 6.32<br>CG: 14.50 ± 5.70	Lawton IADL<br>EG1: 5.50 (3.00, 11.50)<br>EG2: 6.00 (2.75, 9.75)<br>EG3: 5.50 (1.75, 11.00)<br>CG: 6.00 (2.50, 6.00)
Cho et al. (2016) [50]	MMSE score 18-23	Not reported	EG1: 5.1 ± 2.2 mo<br>EG2: 5.9 ± 2.2 mo<br>CG: 6.5 ± 1.5 mo	Not reported	MMSE<br>EG1: 17.8 ± 3.8<br>EG2: 16.9 ± 3.2<br>CG: 18.6 ± 3.4	FIM<br>EG1: 79.1 ± 21.8<br>EG2: 82.4 ± 17.6<br>CG: 76.8 ± 16.3
Faria et al. (2016) [51]	MMSE score ≥ 15	Not reported	EG: 7 (4-49) mo<br>CG: 4 (3-11.5) mo	Not reported	MMSE<br>EG: 23 (20.5-26)<br>CG: 23 (20.5-26)	SIS-ADL<br>EG: 50 (37.5-80.2)<br>CG: 43.8 (14.6-53.1)
Feng et al. (2025) [52]	PSCI without dementia (Expert Consensus on the Management of Cognitive Impairment after Stroke)	EG: H (16), I (14)<br>CG: H (10), I (20)	EG: 132.7 ± 26.5 d<br>CG: 126.3 ± 24.2 d	NIHSS score ≤ 7	MMSE<br>EG: 24 (IQR=2)<br>CG: 24 (IQR=2)	MBI<br>EG: 37 (IQR=1)<br>CG: 38 (IQR=6.3)
He et al. (2021) [53]	MoCA score < 26	EG: H (17), I (15)<br>CG: H (19), I (13)	≤ 6 mo	Not reported	MoCA<br>EG: 14.6 ± 2.6<br>CG: 14.5 ± 2.4	MBI<br>EG: 42 (30, 55)<br>CG: 42 (30, 52)
Ho et al. (2022) [54]	MMSE score 20-26	EG: H (5), I (14)<br>CG: H (11), I (9)	EG: 19.89 ± 18.95 mo<br>CG: 31.78 ± 23.96 mo	Brunnstrom stage (hand)<br>EG: 4.32 ± 1.25<br>CG: 4.08 ± 1.10	MMSE<br>EG: 24.88 ± 2.53<br>CG: 24.94 ± 3.07	SIS-ADL<br>EG: 67.59 ± 18.91<br>CG: 57.83 ± 20.51
Li et al. (2021) [55]	MMSE score ≥ 20	Not reported	EG: 78.2 ± 40 d<br>CG: 69.2 ± 51 d	Brunnstrom stage (hand)<br>EG: 3 (IQR=2)<br>CG: 4 (IQR=3)	MMSE<br>EG: 26 (IQR=4)<br>CG: 25 (IQR=3)	BI<br>EG: 64.67 ± 12<br>CG: 63 ± 13

Supplementary Table S2. *Cont.*

Author(year)	Cognitive impairment criteria or diagnosis	Stroke type	Time since stroke	Stroke severity	Baseline cognitive status	Baseline functional status
Liu et al. (2024) [56]	MMSE score < 20 or MoCA score < 26	Hemorrhagic or ischemic	EG1: 19.5 ± 15.3 mo<br>EG2: 19.3 ± 19.5 mo<br>CG: 39.0 ± 44.2 mo	Not reported	MMSE<br>EG1: 24.6 ± 3.2<br>EG2: 25.0 ± 3.3<br>CG: 24.8 ± 3.2	Lawton IADL<br>Not reported
Liu et al. (2023) [57]	PSCI<br>18 ≤ MoCA score < 26	EG: H (3), I (12)<br>CG: H (6), I (9)	EG: 42.93 ± 34.54 mo<br>CG: 29.27 ± 36.51 mo	Not reported	MoCA<br>EG: 21.47 ± 2.67<br>CG: 21.27 ± 2.76	MBI<br>EG: 64.67 ± 11.41<br>CG: 59.33 ± 10.83
Oliveri et al. (2023) [58]	Not reported	EG: H (5), I (10)<br>CG: H (10), I (6)	EG: 53.5 ± 29.6 d<br>CG: 53.9 ± 29.7 d	Not reported	Attentional matrices<br>EG: 22.7 ± 15.1<br>CG: 13.4 ± 14.7<br>MMSE/MoCA not reported	BI<br>EG: 29.3 ± 25.6<br>CG: 32.1 ± 26.6
Park & Ha (2023) [59]	K-MMSE-2 score 17-27	EG: H (9), I (11)<br>CG1: H (10), I (10)<br>CG2: H (8), I (12)	3-36 mo	Not reported	K-MMSE-2<br>EG: 20.35 ± 1.56<br>CG1: 20.20 ± 1.82<br>CG2: 19.85 ± 1.63	K-MBI<br>EG: 56.80 ± 12.78<br>CG1: 56.20 ± 8.91<br>CG2: 55.05 ± 7.78
Soni et al. (2025) [60]	VCI	EG: H (6), I (19)<br>CG: H (7), I (18)	Not reported	NIHSS<br>EG: 27.56 ± 8.29<br>CG: 27.00 ± 8.55	MoCA[a]<br>Raw baseline range<br>(EG: 13-23, CG: 13-23)	BI[a]<br>Raw baseline range<br>(EG: 20-65, CG: 20-60)<br>Lawton IADL[a]<br>Raw baseline range<br>(EG: 0-4, CG: 0-4)
Veisi-Pirkoohi et al. (2020) [61]	Not reported	Not reported	4-18 mo	Not reported	IVA-2 (Auditory Attention)<br>EG: 74.24 ± 23.61<br>CG: 69 ± 19.07<br>MMSE/MoCA not reported	BI, Luton Index
Yeh et al. (2022) [62]	MMSE score ≥ 19, MoCA score < 26, CDR score ≤ 0.5	EG1: H (12), I (8)<br>EG2: H (10), I (8)<br>CG: H (5), I (12), U (1)	EG1: 54.75 ± 55.24 mo<br>EG2: 49.83 ± 45.43 mo<br>CG: 34.28 ± 31.54 mo	NIHSS<br>EG1: 5.05 ± 4.72<br>EG2: 4.61 ± 3.05<br>CG: 4.5 ± 3.33	MMSE<br>EG1: 25.15 ± 3.83<br>EG2: 25.50 ± 2.81<br>CG: 25.33 ± 3.60,<br>MoCA<br>EG1: 19.15 ± 4.90<br>EG2: 20.28 ± 5.34<br>CG: 19.39 ± 4.33	FIM<br>EG1: 103.80 ± 18.03<br>EG2: 103.44 ± 18.10<br>CG: 109.39 ± 11.21<br>Lawton IADL<br>EG1: 22.65 ± 7.56<br>EG2: 23.45 ± 5.90<br>CG: 24.33 ± 4.61

Supplementary Table S2. *Cont.*

Author(year)	Cognitive impairment criteria or diagnosis	Stroke type	Time since stroke	Stroke severity	Baseline cognitive status	Baseline functional status
Yoo et al. (2015) [63]	Not reported	Not reported	EG: 11.8 ± 7.5 mo<br>CG: 10.7 ± 6.2 mo	Not reported	CNT (Digit Span Test)<br>EG: 3.62 ± 1.35<br>CG: 3.72 ± 1.23	FIM<br>EG: 73.34 ± 20.51<br>CG: 75.56 ± 19.65
He et al. (2021) [64]	MoCA (Fuzhou version) score 10-26	Hemorrhagic or ischemic	EG1: 2 (1, 7) mo<br>EG2: 2 (1, 6.5) mo<br>CG: 1 (1, 3) mo	Not reported	MoCA<br>EG1: 19.22 ± 4.22<br>EG2: 19.17 ± 4.57<br>CG: 16.60 ± 6.04	MBI<br>EG1: 61.39 ± 19.71<br>EG2: 59.88 ± 17.24<br>CG: 51.04 ± 27.08<br>Lawton IADL<br>EG1: 6 (5, 11)<br>EG2: 8 (5.25, 9)<br>CG: 4 (4, 7)
Zhang et al. (2025) [65]	MMSE score > 10	EG: H (11), I (13)<br>CG: H (10), I (14)	EG: 42.5 (IQR=60) d<br>CG: 42.5 (IQR=55.75) d	Not reported	MMSE<br>EG: 17.5 (IQR=9.50)<br>CG: 19 (IQR=8.75)	MBI<br>EG: 43.08 ± 25.20<br>CG: 43.25 ± 25.58

Continuous variables are reported as mean ± SD, median (Q1–Q3), median (IQR), or median (range), as presented in the original studies.

[a] Raw baseline ranges are presented because mean and standard deviation values were reported only as standardized z-scores in the original study.

Reference numbers correspond to those in the main manuscript.

ADL: activities of daily living; BI: Barthel Index; CG: control group; CNT: Computerized Neuropsychological Test; d: days; EG: experimental group; FIM: Functional Independence Measure; H: hemorrhagic; I: ischemic; IAFAI: Adults and Older Adults Functional Assessment Inventory; IADL: instrumental activities of daily living; IQR: interquartile range; IVA-2: Integrated Auditory Visual 2 test; K-MBI: Korean version of the Modified Barthel Index; K-MMSE-2: Korean version of the Mini-Mental State Examination, second edition; Lawton IADL: Lawton Instrumental Activities of Daily Living scale; MBI: Modified Barthel Index; MMSE: Mini-Mental State Examination; mo: months; MoCA: Montreal Cognitive Assessment; NIHSS: National Institutes of Health Stroke Scale; PSCI: post-stroke cognitive impairment; Q1: first quartile; Q3: third quartile; SD: standard deviation; SIS-ADL: Stroke Impact Scale activities of daily living; U: unknown; VCI: vascular cognitive impairment.
