# Supplementary material for: Effects of Digital Cognitive Rehabilitation on Functional Performance After Stroke: A Systematic Review and Meta-Analysis of ADL and IADL Outcomes
Source: Healthcare (Basel). 2026 Aug 2;14(15):2355. doi: 10.3390/healthcare14152355 (PMC13465463; doi:10.3390/healthcare14152355)
Supplement: Supplementary file 1 [file healthcare-14-02355-s001.zip › Table S3. Summary of studies retained in the narrative synthesis but excluded from the meta-analysis.pdf]

Supplementary Table S3. Summary of studies retained in the narrative synthesis but excluded from the meta-analysis.

Author(year)	Outcome domain	Outcome measure	Main narrative finding	Direction of finding
Faria et al. (2016) [51]	ADL	SIS-ADL	Within-group changes in SIS-ADL were not statistically significant in either group, and no significant between-group difference was reported	No significant within- or between-group difference
He et al. (2021) [53]	ADL	MBI	Significant group, time, and group-by-time interaction effects were reported for MBI	Significant effect favoring intervention
Li et al. (2021) [55]	ADL	BI	BI scores improved significantly within both groups, but no significant between-group difference was reported	Within-group improvements without between-group difference
Chen et al. (2024) [49]	IADL	Lawton IADL	A significant between-group difference in IADL change was reported, favoring the combined intervention condition	Significant effect favoring combined intervention
Liu et al. (2024) [56]	IADL	Lawton IADL	No significant between-group difference was reported for IADL	No significant between-group difference
Camara et al. (2025) [48]	ADL/IADL	IAFAI	Statistically significant improvement in a functional performance measure covering both ADL and IADL components was reported, favoring the intervention condition	Significant effect favoring intervention

All six studies were excluded from the meta-analysis because sufficient statistical information for effect-size calculation was not available. ADL: activities of daily living; BI: Barthel Index; IAFAI: Adults and Older Adults Functional Assessment Inventory; IADL: instrumental activities of daily living; MBI: Modified Barthel Index; SIS: Stroke Impact Scale.
